# Cariprazine as adjunctive treatment of catatonia in schizoaffective disorder: a case report.

**DOI:** 10.1192/j.eurpsy.2024.505

**Published:** 2024-08-27

**Authors:** D. Magalhaes, L. Fernandes

**Affiliations:** Mental Health Department, Hospital Prof. Doutor Fernando Fonseca, Lisbon, Portugal

## Abstract

**Introduction:**

Cariprazine is one of the most recent innovations in neuropsychopharmacology, with evidence for its efficacy in affective and psychotic spectrum disorders.

**Objectives:**

To present a case that highlights cariprazine’s potential use outside the regulatory approved indications.

**Methods:**

Case report using CARE guidelines and a narrative review.

**Results:**

We present the case of a 41-year-old male readmitted to a psychiatric inpatient unit due to three months of mutism and withdrawal. At admission, the patient did not communicate verbally or in writing, but he complied with simple orders, and his consciousness remained unimpaired. He scored 11 points on the Bush-Francis Catatonia Rating Scale (BFCRS), indicating immobility, mutism, staring, withdrawal, ambitendency, and automatic obedience. We observed psychomotor retardation and indirect signs of a depressive mood, including the omega sign. His medical history included ongoing psychiatric treatment since the age of 30, with two prior admissions to an acute inpatient unit. At the time of admission, he was treated with olanzapine 20 mg/day, lorazepam 2 mg/day (recently downtitrated), venlafaxine 150 mg/day, and bupropion 150 mg/day. At the start of the current episode, the patient’s diagnosis was uncertain, with previous descriptions of psychotic, affective, and catatonic features. Due to suspicion of catatonia, we administered a high dose of lorazepam (8 mg/day), resulting in a partial response with a 4-point reduction in the BFCRS. We discontinued bupropion, increased venlafaxine to 225 mg, and switched from olanzapine to cariprazine using a taper, washout, and switch strategy. Psychotic symptoms briefly appeared when the patient was not taking a dopamine D2-receptor modulatory drug. We identified mild possible adverse drug reactions, including akathisia, transient insomnia, and daytime sleepiness. At a dose of 6 mg/day of cariprazine, we observed complete remission of catatonia (BFCRS=0) and significant improvement in affective and psychotic symptoms. The patient was discharged home with diagnoses of catatonia and schizoaffective disorder, prescribed 6mg/day of cariprazine, 225mg/day of venlafaxine, and 2,5mg/day of lorazepam. At the 6-month follow-up, the patient continues to exhibit clinical stability.

**Conclusions:**

This case emphasizes the safety and potential effectiveness of cariprazine in treating catatonia within the context of schizoaffective disorder. We consider that the partial agonist properties of cariprazine could theoretically reduce the risk of exacerbating catatonia, a risk typically associated with full D2-receptor antagonists. Other mechanisms of action, such as D3 partial agonism, may also contribute to the improvement or at least the non-aggravation of catatonic symptoms. Cariprazine’s mood-stabilizing properties make it a promising off-label choice for treating schizoaffective disorder, especially when catatonic features are present.

**Disclosure of Interest:**

None Declared

